# CAUSAL EFFECT OF ANTI-DEMENTIA DRUGS ON PATIENTS’ ECONOMIC BURDEN USING DOUBLE/DEBIASED MACHINE LEARNING APPROACH

**DOI:** 10.1093/geroni/igad104.3213

**Published:** 2023-12-21

**Authors:** Xiangxiang Jiang, Gang Lv, Minghui Li, Kevin Lu

**Affiliations:** University of South Carolina, Columbia, South Carolina, United States; First Chinese PLA Hospital, Beijing, Beijing, China (People’s Republic); University of Tennessee, Memphis, Tennessee, United States; University of South Carolina, Columbia, South Carolina, United States

## Abstract

Alzheimer’s disease and related dementia (ADRD) present a significant public health challenge and carry a substantial financial burden. Exploring avenues for treatment, the administration of anti-dementia drugs has emerged as a potential intervention for ADRD. However, prior research has been susceptible to omitted variable bias due to data limitations and struggled to establish causal relationships due to confounding by indication. Our study aims to uncover the causal impact of anti-dementia drug utilization on the economic burden among ADRD patients through Double/Debiased Machine Learning (DML) method. Leveraging data from the Medicare Current Beneficiary Survey (MCBS) spanning 2015 to 2017, we utilized a nationally representative survey linked to Medicare data, providing comprehensive insights. DML techniques, including Random Forest (RF) regression and Least Absolute Shrinkage and Selection Operator (LASSO) regression algorithms, were employed to investigate the causal effect. After exclusion and inclusion, a total of 218,237 ADRD patients used anti-dementia drugs, with an average total medical cost of $16,695.87; 95.51% (4,646,408 patients) of ADRD patients did not use anti-dementia drug, with an average total medical cost of $27,567.09. Results from generalized linear regression showed that anti-dementia drug use did not have a significant impact on total medial cost (β = -0.29, P = 0.10). However, results from DML demonstrated a significant true effect [RF regression (β = -0.85, P = 6.79×10-3), LASSO regression (β = -0.78, P = 9.95×10-3)]. To conclude, our analysis demonstrated the causal effect of anti-dementia drug utilization in reducing overall medical costs borne by ADRD patients.

